# Evaluating the relationship between dental caries number and salivary level of IgA in adults

**DOI:** 10.4317/jced.54271

**Published:** 2018-01-01

**Authors:** Hesam Haeri-Araghi, Mahdieh Zarabadipour, Shadab Safarzadeh-Khosroshahi, Monirsadat Mirzadeh

**Affiliations:** 1 General Dentist, Student Research Committee of Dentistry, Faculty of Dentistry, Qazvin University of Medical Sciences, Qazvin, Iran; 2 Assistant Professor of Oral Medicine, Dental Caries Prevention Research Center, Qazvin University of Medical Sciences, Qazvin, Iran; 3 Assistant Professor of Operative Dentistry, Islamic Azad University, Dental Branch, Tehran, Iran; 4 Assistant Professor of Community Medicine, Qazvin University of Medical Sciences, Qazvin, Iran

## Abstract

**Background:**

Dental caries are the most common mouth infectious disease and also chronic disease of childhood. Saliva plays different roles in oral cavity; for example, salivary immunoglobulins play significant role in body and oral immunity. Various studies were conducted on the different effects of IgA on oral cavity, especially dental caries, and reported controversial results. The current study aimed to compare salivary IgA level at different stages of dental caries in adults.

**Material and Methods:**

A total of 40 adults, aged 20 to 40 years, referred to the department of oral medicine at Qazvin Faculty of Dentistry, were selected voluntarily based on the number of decayed teeth. Their unstimulated saliva was collected by the spitting method. The cases were assigned to 4 groups each of 10, based on the number of decayed teeth, as follows: Group 1: Caries free, Group 2: With 1 or 2 decayed teeth, Group 3: With 3 or 4 decayed teeth, and Group 4: With 5 or more decayed teeth. None of the cases had systemic diseases or the history of using medicines which affect the quality or quantity of saliva. The salivary IgA level of the cases was measured immunoturbidometrically and analyzed by ANOVA and t test.

**Results:**

Significant difference was observed between the groups 1 and 4, but there was no significant difference between the other groups.

**Conclusions:**

According to the results of the current study, the salivary IgA can be considered as an index for the function of immune system, which may be increased by the number of decayed teeth. In fact, the increase of salivary IgA is just the response of immune system to the accumulation of microorganisms and may be the attempt of body to control them.

** Key words:**Saliva, IgA, Dental caries.

## Introduction

Dental caries is the most common infectious disease in human, which is under the influence of different factors such as race, inheritance, nutritional conditions, culture, and personal hygiene (1,2). The saliva, as a vital fluid of mouth, plays an important role in balancing the mouth and it components may affect personal health status. Immunoglobulins are among basic components of saliva (1), and play vital role in the acquired and non-acquired immunity against microorganisms (1,3). There are different types of immunoglobulins (IgA, G, and M) which IgA is the most particular one (60% of all immunoglobulins are IgA) (1). IgA provides immunity through inhibition of adhesion, reduction of hydrophobicity, and excretion of toxins of microorganisms (2-5). Salivary IgA can play a synergistic role with salivary lysozyme and fight oral cavity microbes (2). The effects of IgA on oral cavity, focusing dental decays, were evaluated in different studies, but contradictory results were reported; for example, Koga-ito reported the increase of IgA in adults with lower number of decayed teeth, but did not confirm the same results in children (3). Shifa indicated no significant relationship between the number of decayed teeth and level of IgA, although he reported a little increase in the level of IgA in the caries free people (1). On the other hand, Gornowicz and Fidalgo indicated higher levels of salivary IgA in people with more decayed teeth (4,6).

The current study aimed to evaluate the probable relationship between the level of salivary IgA and the number of decayed teeth.

## Material and Methods

-Study population

All patients, aged 20 to 40 years, who referred to the department of oral medicine at Qazvin University of Medical Sciences for routine dental examination, were examined by oral and maxillofacial specialist for recognizing the number of dental caries (based on ICDAS), additional necessary radiographic examination was prescribed to approve the number of their decayed teeth. Then, after signing the written informed consent, the participants were assigned to 4 groups each of 10, based on the number of decayed teeth as follows: Group 1- Caries free; Group 2- With 1 or 2 decayed teeth; Group 3- With 3 and 4 decayed teeth; Group 4- With 5 or more decayed teeth. The exclusion criteria were as follows: having systemic diseases or using systemic or localized medicines, or consumption of drugs affecting the quality and quantity of saliva, using systemic antibiotics or systemic corticosteroids 2 weeks prior to sampling, history of systemic or particular oral infection 2 weeks prior to sampling, verified periodontitis or other active and progressive periodontal diseases, smoking and alcohol consumption, exercising as professional athletics.

The study protocol was approved by the Ethical Committee of Qazvin University of Medical Sciences, Qazvin, Iran (approval code: IR.QUMS.REC.1395.16).

-Saliva samples collection

Saliva sample collection was performed from 10:00 AM to 12:00 AM. The participants were fasted for 2 hours prior to sample collection and sat in a totally relaxed position for several minutes prior to the sampling. Then, their unstimulated saliva was collected in the sterile tubes within 5 minutes by spitting method. Samples were transferred to the freezer within less than hour and stored at -20°C up to use.

-Analysis of salivary IgA

First, the samples were defrozen and, then, were centrifuged at 4000 rpm for 10 minutes. The total amount of IgA was measured immunoturbidometrically. Immunoturbidometry is a method designed based on different photometric absorbance before and after the reaction between the antibodies sensitized against human IgA available in kit and IgA antigen available in saliva sample and also can determine the concentration of IgA. Samples were codified with symbols, abbreviations, and numbers and the expert who conducted the tests were completely blind for the samples and final results. Data were transferred to SPSS version 21 and analyzed by the statistical tests.

## Results

Out of 40 current study participants, 20 were female and 20 male; the gender distribution of the groups is shown in  Table 1. The Chi-square test results indicated no significant differences between the study groups regarding the gender (*P*-value > 0.05); hence, both groups were similar regarding the gender distribution.

**Table 1:**
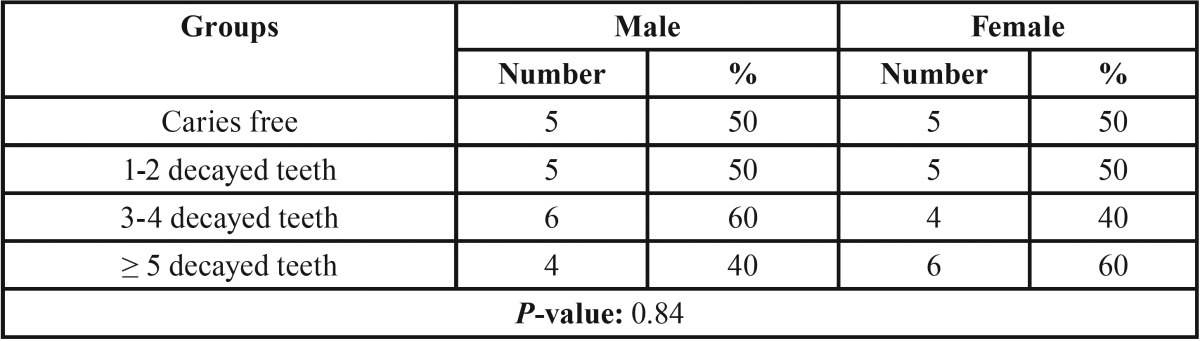
Gender distribution between groups of the study.

After evaluating the normality of data by Kolmogorov-Smirnov test (*P*-value >0.05), the ANOVA was used to evaluate data and differences between the groups, which indicated significant differences between the study groups regarding the level of IgA (*P*-value = 0.046) ( Table 2). The post hoc test results showed a significant difference between some of the study groups regarding the level of salivary IgA. Accordingly, a significant difference was observed between groups 1 and 4, but the difference between other groups was insignificant IgA (*P*-value = 0.027). According to the obtained results, increasing the number of decayed teeth increases the level of salivary IgA in all study groups. Comparison of the level of salivary IgA between genders showed no significant changes in this regard between males and females (*P*-value = 0.051).

**Table 2:**
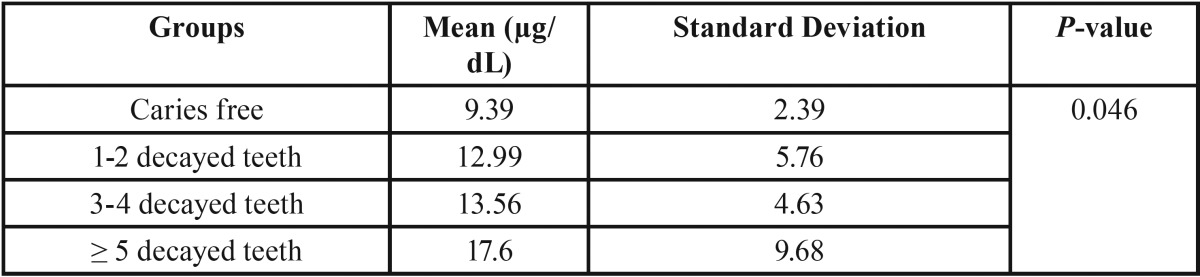
Comparison of salivary IgA levels between groups.

Based on statistical analysis there was a significant positive correlation between dental caries and IgA levels (r=0.51, *p*=0.001).Linear regression was used to determine the ability of dental caries to predict IgA level changes. The test showed that 26 percentage of IgA changes is related to dental caries.

## Discussion

Dental caries cause changes in immune system, due to its infectious nature. The immune response may result in the secretion of IgA in serum and saliva (1,7). The current study was conducted on the saliva samples of 40 cases, aged 20 to 40 years, who referred to the department of oral medicine at Faculty of Dentistry, Qazvin University of Medical Sciences. The participants were assigned to 4 groups, based on the number of decayed teeth, and were assessed for the level of salivary IgA. The secretion of salivary IgA in patients with dental caries was discussed and approved in different studies; for example, a review study by Fidalgo (2014) indicated that dental caries resulted in the increase of salivary IgA level (4). In a study by Shifa it was shown that an immune response against dental decay played a protective role against the progression of caries (1).

According to the analysis of the current study results, increasing the number of decayed teeth (particularly between the groups 1 and 4) significantly increased the amount of salivary IgA, which was consistent with the results of the studies by Fidalgo (4), Ranadheer (2), Gornowicz (6), Bagherian (8) and yang (9). These studies also indicated the higher levels of salivary IgA in the cases with dental caries, compared with caries free ones.

In contrast to the results of the current study, Koga-ito (3) and Kuriakose (10) indicated in their studies that the amount of salivary IgA reduced following the increase in the number of decayed teeth and according to their conclusion, this reduction could be as a result of body defence mechanism. But it seems that the difference between the results of the current study and the aforementioned ones relied on differences in the age, IgA measurement method, sample size, and race. In addition, the study by Shifa (2008) also indicated no significant relationship between the amount of salivary IgA and number of decayed teeth (1); the significant difference between the results of this study and the current one may derive from the fact that Shifa only investigated the children aged 3 to 6 years with primary dentition. As the results of the study showed, there was a positive correlation between high levels of dental caries and salivary levels of IgA. The result is confirmed by bagherian (8) that revealed a weak inverse correlation between the variables.

Nowadays, using saliva as an important marker in diagnosis, treatment, and control of different systemic diseases have met a warm welcome; also many studies reported that sometimes the initiation of salivary changes even occur prior to serum and systemic changes (3,11). Hence, there is a strong tendency toward using saliva to diagnose diseases in the early stages (12,13); in addition, using saliva is an easy and cost-effective method, it is safe and non-invasive for the examiner, and also it does not need extensive equipment and facilities(4,14,15). There are a few and inadequate studies on adult’s salivary IgA and only the studies by Koga-ito (3) and Gornowicz (6) involved adults. Therefore, investigation in this regard seemed to be necessary. In the current study, dental carries index was used to assess the relationship with salivary IgA. Many previously conducted studies used decay-missing-filled (DMF) index. It is noteworthy that assessment of IgA level indicates the response of immune system to microorganisms, but DMFT involves different factors; for example, the question always rises in DMFT index, which is recorded at the time of examination, is that: “Is it true to involve the teeth missed due to reasons other than decay (such as trauma, dental aesthetics, and orthodontic) and/or the teeth filled due to different reasons (such as dental aesthetics, trauma, diastema closure, etc.) in the level of IgA and immune response stimulation?”. Hence, the current study aimed at using dental caries index as a variable influencing the level of salivary IgA.

## Conclusions

According to the results of the current study, by increasing the number of decayed teeth the amount of salivary IgA, as an index for immune system function, increases; this increase is statistically significant compared with the groups with very low number of decayed teeth or caries free. Hence, it seems that the salivary IgA is not a caries prevention factor and is only a response of immune system to the microorganisms or even an effort to control them.
